# Supplementary material for: Maternal dietary folate intake with folic acid supplements and wheeze and eczema in children aged 2 years in the Japan Environment and Children’s Study
Source: PLoS One. 2022 Aug 22;17(8):e0272968. doi: 10.1371/journal.pone.0272968 (PMC9394831; doi:10.1371/journal.pone.0272968)
Supplement: S1 Table — (DOCX) [file pone.0272968.s001.docx]

**S1 Table Characteristics of the participants (mothers and children)**

|  |  | **Wheeze** |  |  |  | **Eczema** |  |  |
| --- | --- | --- | --- | --- | --- | --- | --- | --- |
|  |  | **n (%)** |  |  |  | **n (%)** |  |  |
|  |  | **Yes** |  | **No** |  | **Yes** |  | **No** |
| **Age** | **≤24 years** | **1669 (8.2%)** |  | **5481 (8.5%)** |  | **846 (7.6%)** |  | **6323 (8.6%)** |
|  | **25-34 years** | **13317 (65.2%)** |  | **40377 (62.9%)** |  | **7132 (64.3%)** |  | **46699 (63.4%)** |
|  | **≥35 years** | **5432 (26.6%)** |  | **18307 (28.5%)** |  | **3107 (28.0%)** |  | **20671 (28.1%)** |
| **Education (mother)** | **Junior high/high school/technical college** | **7308 (35.9%)** |  | **22901 (35.8%)** |  | **3646 (33.0%)** |  | **26653 (36.3%)** |
|  | **Professional school/junior college/university/graduate school** | **13044 (64.1%)** |  | **41015 (64.2%)** |  | **7404 (67.0%)** |  | **46760 (63.7%)** |
| **Education (father)** | **Junior high/high school/technical college** | **9560 (47.2%)** |  | **27762 (43.7%)** |  | **4796 (43.6%)** |  | **32670 (44.7%)** |
|  | **Professional school/junior college/university/graduate school** | **10675 (52.8%)** |  | **35831 (56.3%)** |  | **6197 (56.4%)** |  | **40361 (55.3%)** |
| **Allergy (mother)** | **No** | **7612 (37.4%)** |  | **28498 (44.6%)** |  | **3679 (33.3%)** |  | **32507 (44.3%)** |
|  | **Yes** | **12733 (62.6 %)** |  | **35423 (55.4%)** |  | **7372 (66.7%)** |  | **40902 (55.7%)** |
| **Alcohol consumption (during pregnancy)** | **No** | **19589 (96.6%)** |  | **62073 (97.5%)** |  | **10656 (96.9%)** |  | **71201 (97.3%)** |
|  | **Yes** | **680 (3.4%)** |  | **1622 (2.5%)** |  | **341 (3.1%)** |  | **1961 (2.7%)** |
| **Parity** | **Primipara** | **7346 (36.2%)** |  | **28312 (44.5%)** |  | **4408 (40.1%)** |  | **31302 (42.9%)** |
|  | **Multipara** | **12922 (63.8%)** |  | **35257 (55.5%)** |  | **6578 (59.9%)** |  | **41746 (57.1%)** |
| **Body mass index (before pregnancy)** | **<18.5** | **3164 (15.5%)** |  | **10501 (16.4%)** |  | **1812 (16.3%)** |  | **11889 (16.1%)** |
|  | **≥18.5 and <25.0** | **15033 (73.6%)** |  | **47343 (73.8%)** |  | **8214 (74.1%)** |  | **54278 (73.7%)** |
|  | **≥25.0 and <30.0** | **1702 (8.3%)** |  | **4877 (7.6%)** |  | **823 (7.4%)** |  | **5784 (7.8%)** |
|  | **≥30.0** | **515 (2.5%)** |  | **1437 (2.2%)** |  | **235 (2.1%)** |  | **1732 (2.4%)** |
