# Supplementary material for: Maternal dietary folate intake with folic acid supplements and wheeze and eczema in children aged 2 years in the Japan Environment and Children’s Study
Source: PLoS One. 2022 Aug 22;17(8):e0272968. doi: 10.1371/journal.pone.0272968 (PMC9394831; doi:10.1371/journal.pone.0272968)
Supplement: S2 Table — †Adjusted is adjusted for maternal age, sex of child, fetus week number, education (mother and father), allergy (mother), smoking (during pregnancy), alcohol consumption (during pregnancy), body mass index (before pregnancy), parity, breastfeeding term, nursery school and baby farm, omega-3 fatty acid intake (< 1.6 g/day, ≥ 1.6 g/day), vitamin D intake (< 8.5 μg/day, ≥ 8.5 μg/day). The intake of omega-3 fatty acid and vitamin D was classified based on the maternal standard issued by the Ministry of Health, Labor, and Welfare in Japan (2020). ‡OR, odds ratio; §CI, confidence interval. (DOCX) [file pone.0272968.s002.docx]

**S2 Table Folic acid (supplement and diet) and the allergies adjusted with omega-3 fatty acid and vitamin D intake**

|  |  |  |  | **Wheeze** | | **Eczema** | |
| --- | --- | --- | --- | --- | --- | --- | --- |
|  |  |  |  | **Adjusted**^†^ | | **Adjusted**^†^ | |
| **Folic acid supplement** | **Dietary folate (µg/day)** | **n** | **(%)** | **OR**^‡^ | **95% CI**^§^ | **OR**^‡^ | **95% CI**^§^ |
| **No use** | **< 240** | **26,227** | **30.8** | **.966** | **0.918–1.017** | **.895** | **0.840-0.954*** |
|  | **≥ 240 and < 480**  **≥ 480** | **18,734** | **22.0** | **Ref** |  | **Ref** |  |
|  |  | **2,933** | **3.4** | **1.087** | **0.987–1.196** | **1.005** | **0.892–1.132** |
| **Sometimes use** | **< 240** | **9,650** | **11.3** | **.991** | **0.928–1.058** | **1.031** | **0.953–1.116** |
|  | **≥ 240 and < 480** | **7,634** | **9.0** | **1.003** | **0.939–1.072** | **1.011** | **0.933–1.095** |
|  | **≥ 480** | **1,084** | **1.3** | **1.114** | **0.959–1.293** | **.946** | **0.784–1.142** |
| **Daily use** | **< 240** | **9,383** | **11.0** | **.918** | **0.858–0.982*** | **.925** | **0.852–1.004** |
|  | **≥ 240 and < 480** | **8,172** | **9.6** | **.976** | **0.914–1.042** | **.991** | **0.916–1.073** |
|  | **≥ 480** | **1,297** | **1.5** | **1.080** | **0.940–1.242** | **.972** | **0.819–1.154** |

^†^**Adjusted is adjusted for maternal age, sex of child, fetus week number, education (mother and father), allergy (mother), smoking (during pregnancy), alcohol consumption (during pregnancy), body mass index (before pregnancy), parity, breastfeeding term, nursery school and baby farm,** **omega-3 fatty acid intake (< 1.6 g/day, ≥ 1.6 g/day), vitamin D intake (< 8.5 µg/day, ≥ 8.5 µg/day). The intake of omega-3 fatty acid and vitamin D were classified based on the maternal standard issued by the Ministry of Health, Labor, and Welfare in Japan (2020).** ^‡^**OR, odds ratio;** ^§^**CI, confidence interval**
